# Temporal regulation of HIF-1 and NF-κB in hypoxic hepatocarcinoma cells

**DOI:** 10.18632/oncotarget.3352

**Published:** 2015-03-19

**Authors:** Yuan Jiang, Ying Zhu, Xinxin Wang, Juan Gong, Chunyan Hu, Bo Guo, Bo Zhu, Yongsheng Li

**Affiliations:** ^1^ Institute of Cancer, Xinqiao Hospital, Third Military Medical University, Chongqing 400037, China; ^2^ Center for Experimental Therapeutics and Reperfusion Injury, Department of Anesthesia, Perioperative and Pain Medicine, Brigham and Women's Hospital and Harvard Medical School, Boston, MA 02115, USA

**Keywords:** hypoxia, NF-κB, HIF-1, miRNA

## Abstract

Regulations between NF-*κ*B and HIF-1 have not been adequately addressed in previous research. Here, we report that hypoxia increased NF-κB in hepatocellular carcinoma cells. The HIF-1 protein level was rapidly induced by protein stabilization (by 2 hours) and then moderately decreased, whereas mRNA levels were reciprocally increased. We also found that NF-κB p50 and p65 (RelA), but not c-Rel, bound the HIF-1a promoter, thus increasing its transcription. In contrast, miR-199a-5p and miR-93, c-Rel downstream targets, decreased HIF-1α at both the mRNA and protein levels. Dicer1, a key enzyme in miRNA biogenesis, was decreased by acute hypoxia but was later increased by HIF-1, rather than by the above-mentioned NF-κB subunits. Thus, NF-κB both positively and negatively fine-tuned HIF-1 in hypoxic hepatocarcinoma cells.

## INTRODUCTION

Hypoxia, a common feature of all solid tumors, plays essential roles in tumor initiation and development [1]. A key development in the understanding of oxygen-sensing processes and of the mechanisms that regulate the cellular and tissular response to hypoxia came with the discovery of hypoxia-inducible factor-1 (HIF-1) which consists of an oxygen regulated *α*-subunit and a constitutive *β*-subunit [2]. In normoxia, hydroxylation by specific hydroxylases leads to the ubiquitination of the HIF-1α proteins and/or the inhibition of the activity of HIF-1 [3, 4]. Under hypoxia, molecular oxygen is not available for hydroxylation; thus, HIF-1α can accumulate in the cell, translocate into the nucleus, bind with HIF-1β, and promote the transcription of downstream target hypoxia responsive genes (HRGs) thereby playing a central role in tumorigenesis, including energy metabolism, angiogenesis, proliferation and metastasis [5–7].

However, HIF-1 does not mediate the tumor response to hypoxia independently. Cross-talk between HIF-1 and nuclear factor *κ*B (NF-*κ*B) has been previously elucidated. Activation of NF-*κ*B elevates mRNA and protein levels of HIF-1α in multiple cell lines [8, 9]. HIF-1 also promotes NF-*κ*B expression and transcription activity [10–13]. Understanding the correlation between HIF-1 and NF-*κ*B under hypoxia may be of great importance in therapeutically targeting hypoxic tumorigenesis.

To date, five subunit members of NF-κB have been identified in mammals: RelA (p65), c-Rel, RelB, p50 and p52. Among them, characteristics and biofunctions of p50, p65 and c-Rel have been best elucidated [14]. In the present study, we aimed to evaluate the temporal expression of HIF-1 and NF-κB subunits in hepatocellular carcinoma cells (HCC) under short-term and prolonged hypoxia and revealed the underlying regulatory mechanism. Our findings identified a novel molecular signaling network that NF-κB regulates HIF-1 in hypoxic HCC.

## RESULTS

### Differential expression of HIF-1 and NF-*κ*B in HCC under short-term versus prolonged hypoxia

**Figure 1:**
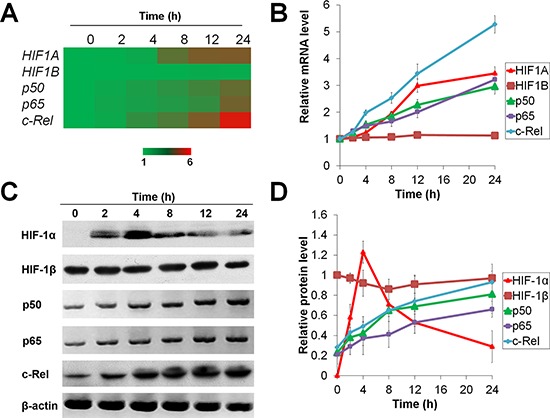
Temporal expression of HIF-1α under short-term and prolonged hypoxia HepG2 cells were exposed to 1% O_2_ for 0-24 h and the mRNA **(A, B)** and protein **(C, D)** expressions of HIF-1α, HIF-1β, NF-κB-p50, p65 and c-Rel were assessed. The results were expressed as the means (A) or means±SEM (B and D) of *n* = 4 independent experiments and normalized with β-actin (*ACTB*).

We first monitored the expressions of HIF-1 and NF-κB in HepG2 and Huh7 cells upon hypoxia (1% O_2_) exposure for 0-24 h *in vitro*. The mRNA levels of *HIF1A*, *NF-κB-p50*, *p65*, and *c-REL* as well as the protein levels of p50, p65 and c-Rel increased in a time-dependent manner (Figure 1A, 1B and S1A). However, the level of HIF-1*α* protein substantially increased following hypoxia treatment for 0–4 h, and decreased subsequently under prolonged hypoxia (Figure 1C, 1D and S1B). These results demonstrate that HIF-1 and NF-κB are temporally and differentially regulated under short-term versus prolonged hypoxia.

### NF-κB temporally modulates HIF-1 expression

**Figure 2:**
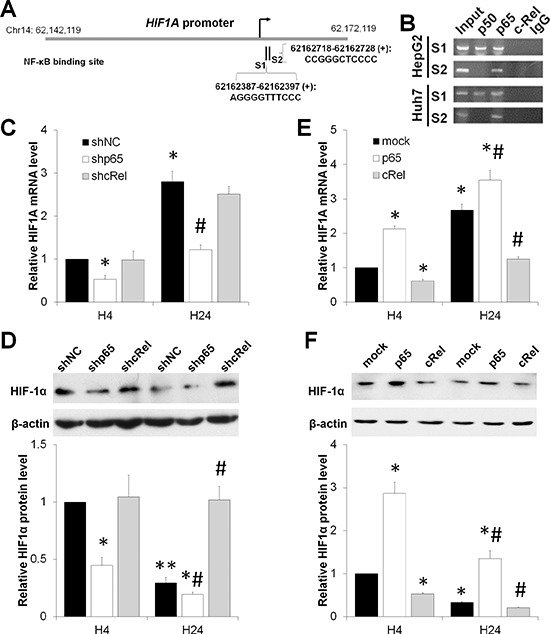
NF-κB dual-regulates HIF-1α under short-term versus prolonged hypoxia **(A)** Vista predictions of NF-κB binding sites on the promoter of *HIF1A*. **(B)** ChIP analysis of the *HIF1A* promoter with NF-κB subunit antibodies and control IgG. **(C,D)** After HepG2 cells were transfected with shNC, shp65 or shcRel, and exposed to hypoxia for 4 h (H4) or 24 h (H24), the mRNA (C) and protein (D) levels of HIF-1α were assessed. The results were expressed as the mean±SEM of four independent experiments. *P < 0.05 and **P < 0.01 compared with shNC plus H4; ^#^*P* < 0.05 compared with shNC plus H24. (E,F) After HepG2 cells were transfected with mock, p65 or cRel, and exposed to hypoxia for 4 h (H4) or 24 h (H24), the mRNA (C) and protein (D) levels of HIF-1α were assessed. The results were expressed as the mean±SEM of four independent experiments. *P < 0.05 and **P < 0.01 compared with mock plus H4; ^#^*P* < 0.05 compared with mock plus H24.

Previous reports indicated that NF-*κ*B enhanced HIF-1 expression and activation [15, 16]. Employing Vista Software [17], we found that both p50 and p65, but not c-Rel, were predicted to directly bind the promoter of *HIF1A* (Figure 2A). Chromatin immunoprecipitation (ChIP) assay also identified these NF-κB binding sites at 62162387-62162397 (for p50 and p65) and 62162718-62162728 (for p65) on the promoter of *HIF-1A*, respectively (Figure 2B).

The findings that p50 and p65 share a mutual site (S1 as shown in Figure 2A and 2B) and the differential expression of HIF-1*α* and NF-*κ*B, especially the reduction of HIF-1*α* level under prolonged hypoxia (Figure 1), led us to question whether a negative regulatory mechanism exists. In contrast to p50 and p65, the subunit p50 does not contain the transactivation dormain (TAD) in the C-terminal [14]. Therefore, we performed knockdown and overexpression of p65 and c-Rel in HepG2 cells and exposed these cells to short-term (4 h) or prolonged (24 h) hypoxia (1% O2) (Figure S2). Consistent with the results from the ChIP assay (Figure 2B), deficiency of p65 significantly reduced the mRNA and protein levels of HIF-1α (Figure 2C and 2D). Neither the mRNA levels of *HIF1A* at both 4 h and 24 h, nor the protein level of HIF-1*α* at 4 h was significantly altered by c-Rel knockdown. Of interest, the HIF-1*α* protein level at 24 h upon hypoxia exposure in shcRel transfected HepG2 cells was significantly upregulated compared with that in shNC group at the same time point (Figure 2C and 2D). Overexpression of p65 in HepG2 cells boosted the mRNA and protein levels of HIF-1*α* in response to hypoxia for 4 or 24 h. In contrast, c-Rel overexpression significantly impaired HIF-1*α* under both short-term and prolonged hypoxia, compared with the mock group (Figure 2E and 2F). Together these findings suggest that the NF-*κ*B subunits p50 and p65 promote HIF-1α transcription, while c-Rel accelerates the reduction of HIF-1α under hypoxia.

### miR-93 and miR-199a-5p, two NF-κB c-Rel downstream miRs, degradate HIF-1α in hypoxic HCC

**Figure 3:**
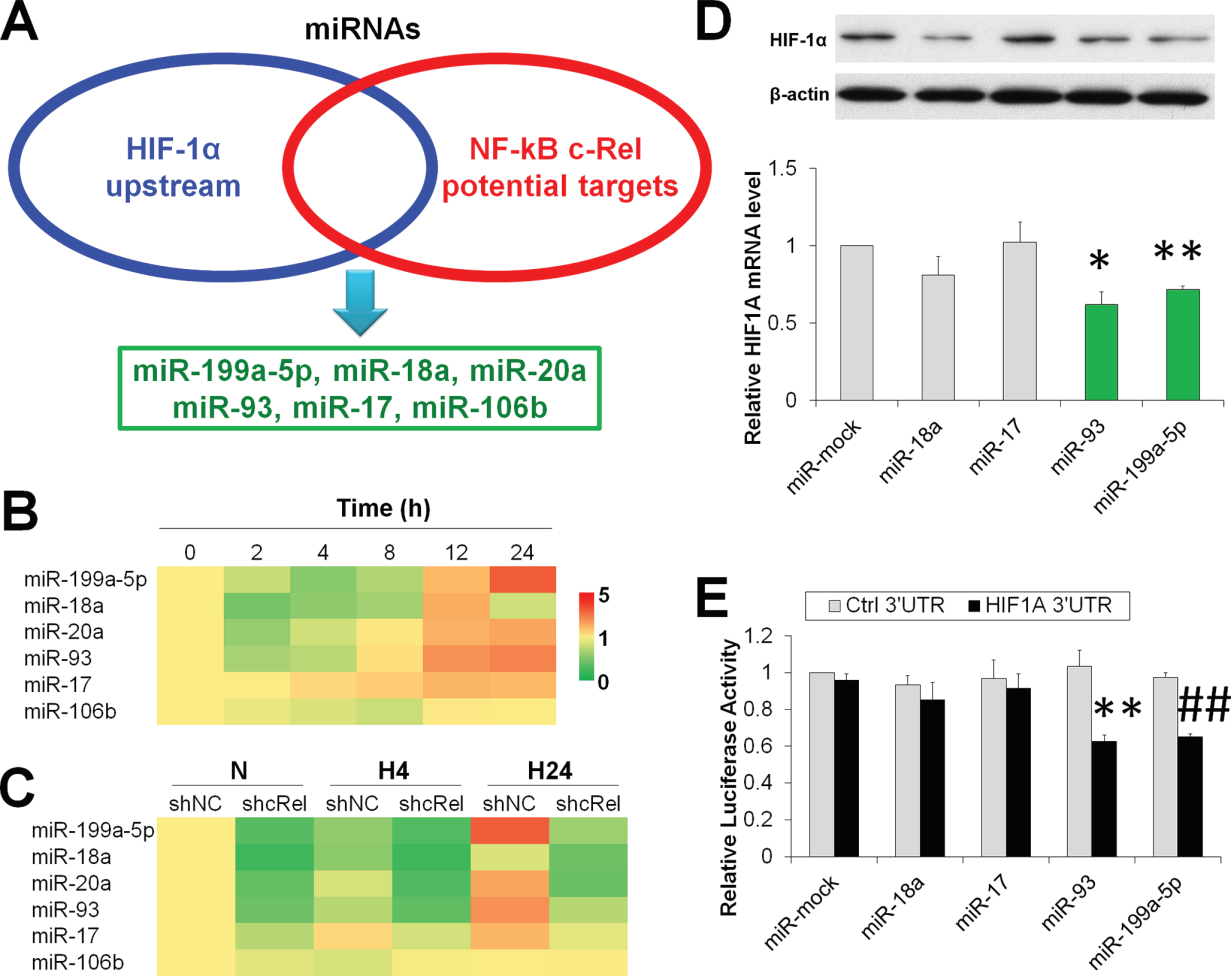
NF-κB downstream miRs suppress HIF-1α expression in hypoxic HCC **(A)** Overlap of the sets of HIF-1α upstream and c-Rel downstream miRNAs. **(B)** Expressions of miR-199a-5p, miR-18a, miR-20a, miR-93, miR-17, and miR-106b in HepG2 cells upon hypoxia challenge for 0-24 h. The results were expressed as the means of four independent experiments and normalized against time 0. (B) After HepG2 cells were transfected with shNC or shcRel, and exposed to hypoxia for 0 h (N), 4 h (H4) or 24 h (H24), the expressions of miR-199a-5p, miR-18a, miR-20a, miR-93, miR-17, and miR-106b were assessed. The results were expressed as the means of four independent experiments and normalized against shNC plus N. **(D)** After HepG2 cells were transfected with miR-mock, miR-18a, miR-17, miR-93, or miR-199a-5p, and exposed to hypoxia for 4 h, the protein and mRNA expressions of HIF-1α were assessed. The results were expressed as the means of four independent experiments and normalized against the miR-mock transfected group. *P < 0.05 and **P < 0.01 compared with miR-mock. **(E)** After HepG2 cells were co-transfected HIF1A 3′UTR or control 3′UTR with miR-mock, miR-18a, miR-17, miR-93, or miR-199a-5p for 48 h, the cells were assessed *via* luciferase assay. The results were normalized against miR-mock plus Ctrl 3′UTR group. **P < 0.01 compared with miR-93 plus Ctrl 3′UTR; ^##^*P* < 0.01 compared with miR-199a-5p plus Ctrl 3′UTR.

miRNAs are non-coding small RNAs that contain 18-24 bp and generally downregulate targets at both mRNA and protein levels posttranscriptionally, i.e., *via* binding the 3′ untranslated region (3′UTR) of target genes [18]. Because the hydroxylases are inactive without enough oxygen molecules [7], we hypothesized whether the underlying mechanism by which c-Rel suppressed HIF-1α was *via* its downstream miRNA(s). Using *in silico* analysis by Targetscan 6.2 [19] and miRGene 2.0 [20], we overlapped the sets of the conserved upstream miRNAs of HIF-1α and the potential downstream target miRNAs of c-Rel. We identified 6 potential candidate miRNAs, including miR-199a-5p, miR-18a, miR-20a, miR-93, miR-17 and miR-106b (Figures 3A and S3). It is noteworthy that there were also potential binding sites for p50/p65 and HIF-1 in the promoter of miR-199a-5p (Figure S3B).

The temporal expressions of these candidate miRNAs under hypoxia were determined (Figure 3B). The level of miR-17 was steadily increased upon hypoxia exposure for 0-24 h, whereas miR-199a-5p, miR-20a, miR-93 and miR-106b decreased in the short-term but increased at prolonged interval. miR-18a was reduced at 2-8 h and 24 h, and it was enhanced at 12 h. These results indicate that most candidate miRNAs were suppressed upon acute hypoxia exposure, while they were enhanced at prolonged intervals.

To validate whether these miRNAs were downstream of c-Rel, we assessed their expression in shNC or shcRel transfected HCC. We found that these miRNAs, except miR-106b, were significantly downregulated by c-Rel knockdown under both normoxia and hypoxia (Figure 3C).

We sought to explore the major miRNAs that contributed to HIF-1α downregulation under prolonged hypoxia. Because most of the above candidate miRNAs were enhanced (Figure 3B) but HIF-1α was dampened at the prolonged intervals (Figure 1C and 1D), we overexpressed miR-18a, miR-17, miR-93, and miR-199a-5p in HepG2 cells and determined the mRNA and protein levels of HIF-1α after cells were exposed to hypoxia for 4 h. Intriguingly, miR-93 and miR-199a-5p significantly suppressed HIF-1α at the mRNA and protein levels. miR-18a reduced the protein levels of HIF-1α, but it did not change the *HIF1A* mRNA amount (Figure 3D). To further evaluate these candidate miRNAs, we assessed the effect of 3′UTR activity on the overexpression of miRNAs. According to the qPCR results, both miR-93 and miR-199a-5p significantly reduced the luciferase activity of *HIF-1A* 3′UTR (Figure 3E). These findings collectively demonstrate that miR-93 and miR-199a-5p, two c-Rel downstream miRNAs, are required for HIF-1α degradation under prolonged hypoxia.

### Dual regulation of Dicer1 and miR-93/199a-5p under hypoxia

**Figure 4:**
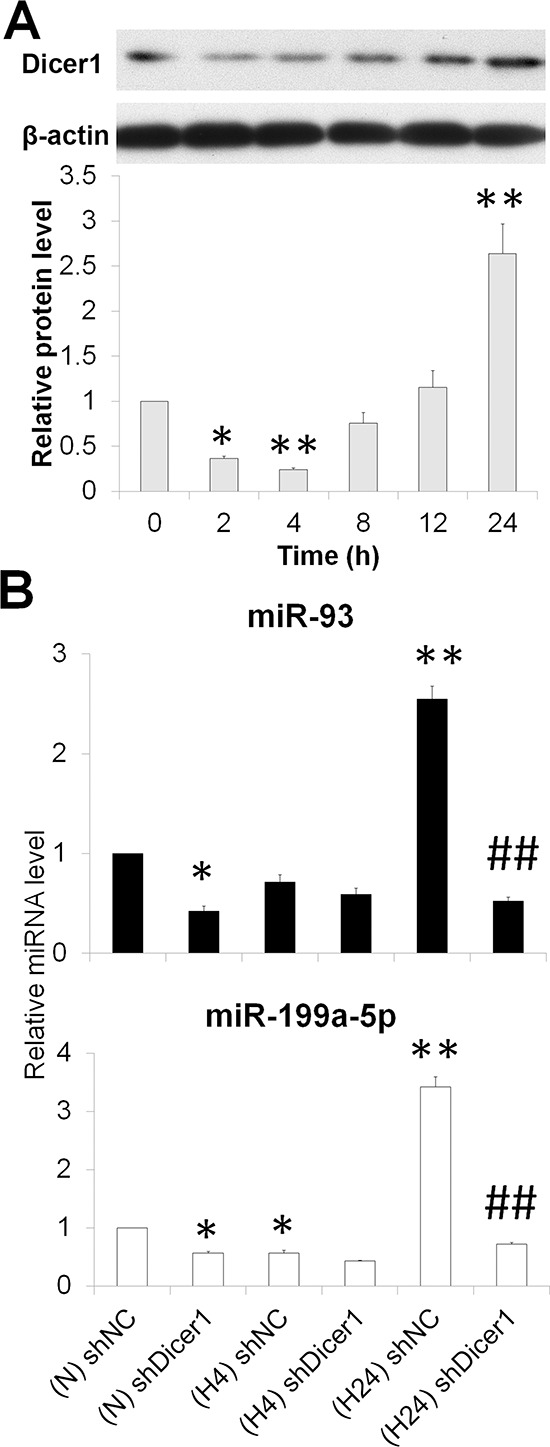
Hypoxia represses miR-93 and miR-199a-5p by downregulating Dicer1 **(A)** The temporal expression of the Dicer1 protein in HepG2 cells under hypoxia for 0-24 h. **(B)** After HepG2 cells were transfected with shNC or shDicer1, and exposed to hypoxia for 0 h (N), 4 h (H4) or 24 h (H24), the levels of miR-93 and miR-199a-5p were assessed. The results were expressed as the mean±SEM of four independent experiments. *P < 0.05 and **P < 0.01 compared with shNC plus N; ^##^*P* < 0.01 compared with shNC plus H24.

Dicer1, also known as endoribonuclease Dicer or helicase with an RNase motif, is an enzyme required for the maturation of the vast majority of miRNAs [21]. We observed a decreased expression level in Dicer1 protein under acute hypoxia, and a subsequently increased level at the prolonged intervals (Figure 4A). Knockdown of Dicer1 downregulated miR-93 and miR-199a-5p in normoxic and prolonged hypoxic HepG2 cells (Figures 4B and S4). These results indicate that acute hypoxia exposure decreased Dicer1 which contributed, at least partially, to the generation of miR-93 and miR-199a-5p.

### Dicer1 promotes HIF-1α degradation under prolonged hypoxia

**Figure 5:**
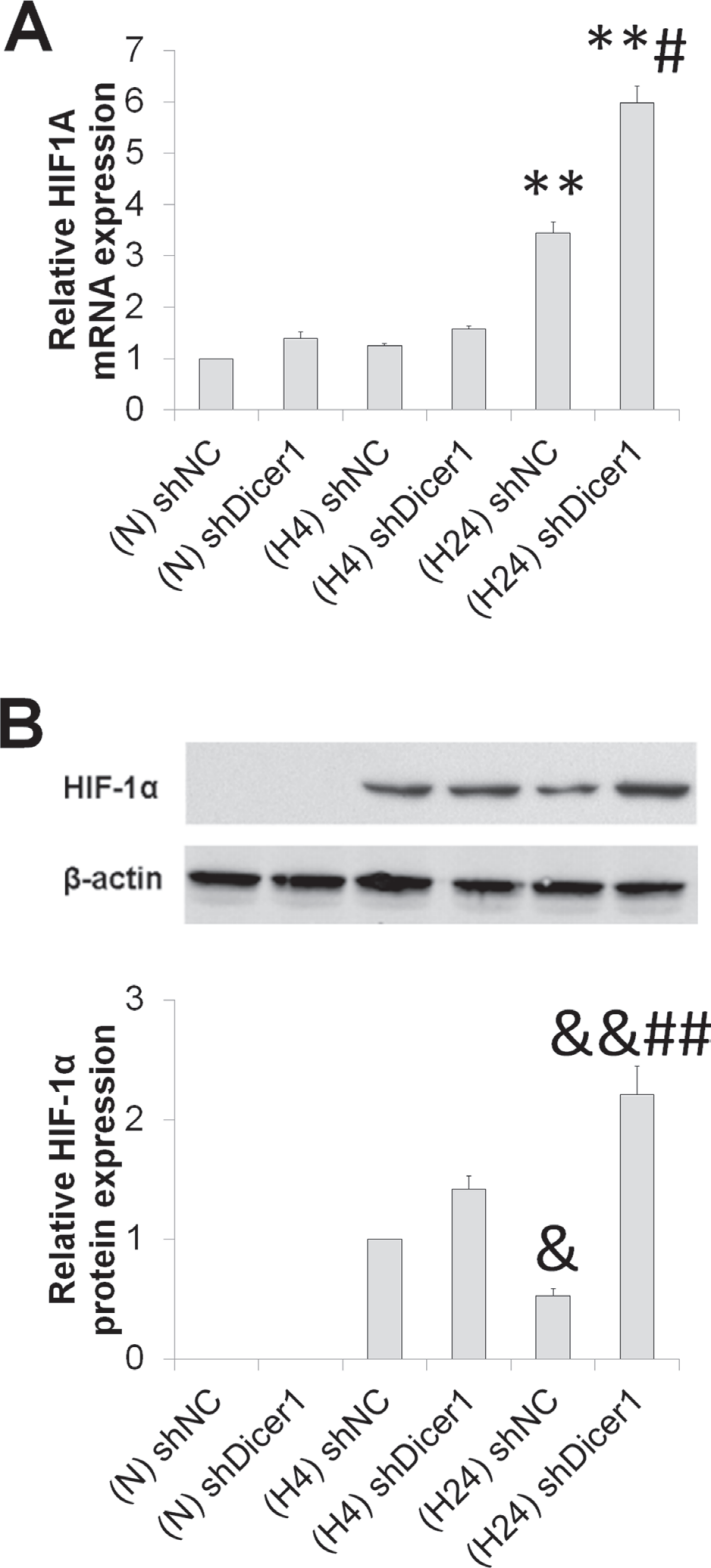
Dicer1 contributes to HIF-1α degradation under prolonged hypoxia After HepG2 cells were transfected with shNC or shDicer1, and exposed to hypoxia for 0 h (N), 4 h (H4) or 24 h (H24), the mRNA **(A)** and protein **(B)** levels of HIF-1α were assessed. The results were expressed as the mean±SEM of four independent experiments. **P < 0.01 compared with shNC plus N; ^&^*P* < 0.05 and ^&&^*P* < 0.01 compared with shNC plus H4; ^##^*P* < 0.01 compared with shNC plus H24.

To address whether the enhanced HIF-1 degradation under prolonged hypoxia resulted from the Dicer1-miRNA axis, we assessed the mRNA and protein levels of HIF-1α in shNC and shDicer1 transfected HCC after exposure to hypoxia for 0, 4 and 24 h. Dicer1 deficiency resulted in enhanced HIF-1α expression at both mRNA and protein levels in the prolonged hypoxic HCC, while no significant change was observed in normoxic and short-term hypoxic HCC (Figure 5). These data demonstrate that Dicer1 contributes to HIF-1α degradation under prolonged hypoxia.

### HIF-1 upregulates Dicer1, miR-93 and miR-199a-5p under hypoxia

**Figure 6:**
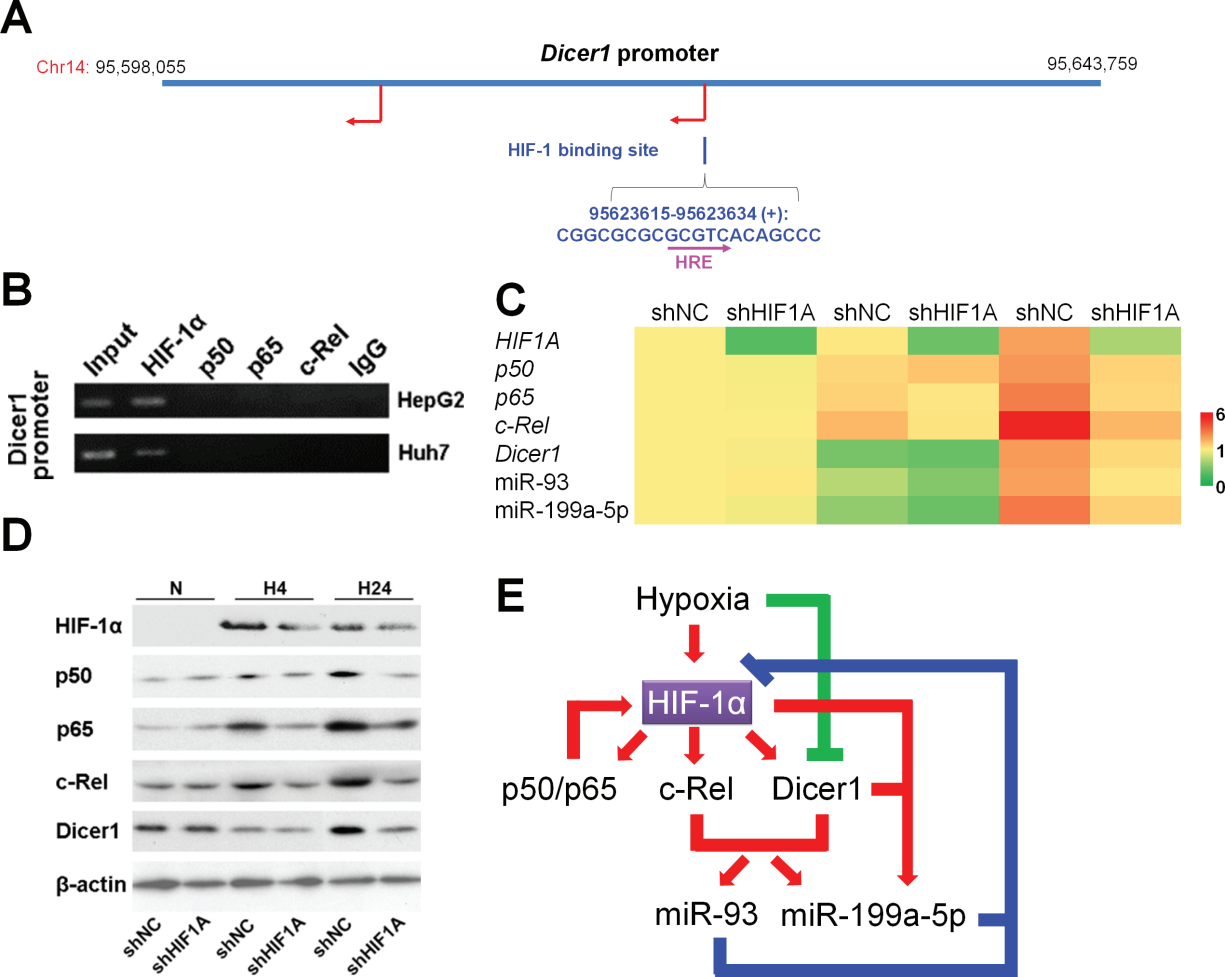
HIF-1 upregulates Dicer1 and miR-93/199a-5p under hypoxia **(A)** Vista prediction of HIF-1 binding sites on the promoter of *Dicer1*. The pink arrow shows the HRE sequence. **(B)** ChIP analysis of the *Dicer1* promoter with NF-κB subunit antibodies and control IgG. **(C,D)** After HepG2 cells were transfected with shNC or shHIF1A, and exposed to hypoxia for 0 h (N), 4 h (H4) or 24 h (H24), the mRNA (C) and protein (D) levels of HIF-1α, p50, p65, cRel, and Dicer1, as well as the expressions of miR-93 and miR-199a-5p (C) were assessed. The results of (C) were expressed as the means of four independent experiments and normalized against shNC plus N. **(E)** The regulatory network involving HIF-1, NF-κB, Dicer1 and miRNAs under hypoxia.

We next questioned whether the elevation of Dicer1 and candidate miRNAs occurs due to regulation by the transcriptional factors HIF-1 and NF-κB. Using Vista software, a binding site of HIF-1 including a hypoxia responsible element (HRE) encoding 5′-GCGTC-3′ was predicted in the promoter of Dicer1 (Figure 6A). No potential binding sites in the *Dicer1* promoter were predicted for p50, p65 and c-Rel, which was also confirmed by the ChIP assay (Figure 6A and 6B). Moreover, the binding sites for HIF-1 were also predicted in the promoters of p50, p65 and c-Rel (Figure S5). Indeed, knockdown of HIF-1α significantly reduced the expressions of p50, p65, c-Rel, Dicer1, miR-93, and miR-199a-5p under hypoxia (Figure 6C and 6D). Together, these results suggested that NF-κB temporally modulates HIF-1 in the short-term versus prolonged hypoxia (Figure 6E).

## DISCUSSION

HIF-1 and NF-κB are both essential for the processes of tumor growth, expansion, invasion, and metastasis. However, the correlation and regulatory mechanisms between them are not yet fully elucidated. In the present study, we demonstrated a regulatory network involving HIF-1, NF-κB, and miRNAs in hypoxic hepatocarcinoma HepG2 and Huh7 cells. Hypoxia initiated HIF-1, which promoted the expression of NF-κB p50, p65, c-Rel and Dicer1. The elevated p50 and p65 boosted HIF-1α, while c-Rel and Dicer1 initiated the generation of miR-93 and miR-199a-5p which were the upstream miRNAs of HIF-1α and contributed to HIF-1α degradation under prolonged hypoxia. The novel regulation of HIF-1 by NF-κB under acute and prolonged hypoxia highlighted new therapeutic options for early cancer intervention.

Elevated HIF-1 and NF-κB are key markers for hypoxic cancer and poor prognosis. Interestingly, our results showed differential expression patterns of HIF-1 and NF-κB in HCC under acute versus prolonged hypoxia. The NF-κB subunits p50 and p65 have been shown to directly interact with the HIF-1*α* promoter [8, 9]. In the present study, we identified that HIF-1 also transcripts *p50*, *p65* and *c-Rel*, suggesting a positive feedback under hypoxia. Most importantly, our results delineated that HIF-1 decreased in HCC at the delayed hypoxia stage, which was supported by few previous reports. For instance, in U2OS (human osteosarcoma) cells, the accumulation of the PHD2 and PHD3 proteins by hypoxia led to accelerated HIF-1 degradation after reoxygenation, while HIF-1 translation in RKO (human colon carcinoma) cells was inhibited by a decrease in GSK3*β* under prolonged hypoxia [22, 23]. Hypoxia can also stimulate HIF-1α SUMOylation and negatively regulate HIF-1 activity by inducing E3 ligases [24]. However, these studies did not provide direct evidence for whether HIF-1 reduction under prolonged hypoxia resulted from enhanced degradation.

Although hydroxylases are mostly disabled to mediate the degradation of HIF-1α, the accumulation of PHDs was enhanced by induced HIF-1 under hypoxia. As discussed above, the elevated PHDs could ensure rapid degradation under re-oxygenation [22]. On the other hand, some scientists proposed that PHDs do not lose their activity completely, and thus the increased PHDs can compensate for their low activity to limit HIF-1 accumulation in prolonged hypoxia [25, 26]. It is noteworthy that hypoxia-enhanced mTOR (Target of rapamycin) can stimulate the translation of HIF-1α, while HIF-1 inhibits the mTOR pathway, forming a negative feedback loop [27]. Indeed, rapamycin can specifically block HIF-1 and HIF-1 dependent transcription via the mTOR pathway [27–29]. Therefore, the regulatory mechanism of the HIF-1 pathway under hypoxia is fine-tuned by several feedback loops at various levels involving stabilization, degradation, and transcription, which remains to be addressed and is of interest.

In the present study, although p50 and p65 contributed to the increase of HIF-1α under hypoxia, another NF-κB subunit c-Rel could not bind the promoter of the *HIF1A* gene. In contrast, it mediated the transcription of miR-93 and miR-199a-5p, two upstream miRNAs that degradate HIF-1α by binding to its conserved 3′UTR. Interestingly, these miRNAs were reduced under acute hypoxia and were subsequently upregulated. The reduction under short-term hypoxia resulted from the hypoxia-decreased Dicer1, which was consistent with a recent report [30]. Of note, the reduction of these miRNAs may also result in enhanced HIF-1 stability under acute hypoxia. Interestingly, Dicer1 was later restored and increased at prolonged intervals of hypoxia. Therefore c-Rel promoted miR-93 and miR-199a-5p transcription and Dicer1 enhanced their dicing, which led to the elevated maturation of these miRNAs and accelerated HIF-1α degradation under prolonged hypoxia.

In summary, our results showed a novel mechanism underlying the regulation of HIF-1 by NF-κB under prolonged hypoxia: the sharp increase of HIF-1 during the acute phase contributes to the elevation of NF-κB and Dicer1. The NF-κB subunits p50 and p65 feedforward enhance HIF-1α transcription, whereas c-Rel downstream miRNAs, including miR-199a-5p and miR-93, together with the HIF-1-elevated Dicer1 contributed to posttranscriptional repression of HIF-1α by targeting its 3′UTRs. Temporal targeting of this regulon in the early hypoxic microenvironment may be particularly essential for effective cancer intervention.

## MATERIALS AND METHODS

### Chemicals and plasmids

All biochemicals and enzymes were of analytical grade and from commercial suppliers. *β*-actin antibody was obtained from Sigma-Aldrich (St. Louis, MO, USA). Lipofectamine 2000 and Trizol Reagents were bought from Invitrogen (Carlsbad, CA, USA). Oligo dT primer, moloney murine leukemia virus (M-MLV) reverse transcriptase, the set of dNTPs and RNase inhibitor were purchased from Promega (Madison, WI, USA). The primers used are listed in Supplementary Table 1 and purchased from Invitrogen (Carlsbad, CA, USA) or Qiagen (Germantown, MD, USA). SYBR Green I PCR Master Mix was the product of Applied Biosystems (Foster City, CA, USA). The β-actin, Dicer1, p50, p65 and c-Rel antibodies were from Cell Signaling (Beverly, MA, USA). The primary antibodies HIF-1α and HIF-1β were from BD Biosciences (San Jose, CA, USA). The jetPRIME^®^ reagent was purchased from PolyPlus Transfection (Illkirch, France). Lentiviral vectors (as listed in Supplementary Table 2) of negative controls (shNC), shHIF1A, shp65, shc-Rel, mock, p65, and c-Rel were from Origene (Rockville, MD, USA); mock-miR, miR-18a, miR-17, miR-199a-5p, miR-93 were obtained from Applied Biological Materials Inc. (Richmond, Canada). 3′UTR luciferase vectors of the vehicle control and *HIF1A* 3′UTR were from Origene (Rockville, MD, USA). Superlight luciferase reporter gene assay kit was from Bioassay Systems (Hayward, CA, USA). ChIP assay kits were from SABioscience (Valencia, CA, USA).

### Cell culture

Human hepatocellular carcinoma HepG2 and Huh7 cells were obtained from American Type Culture Collection (ATCC). Cells were cultured in DMEM (Gibco, Karlsruhe, Germany) supplemented with 10% (v/v) fetal calf serum (Gibco), 2 mM glutamine (Gibco), 50 i.u./ml penicillin and 50 μg/ml streptomycin (Sigma, Deisenhofen, Germany). All cells were maintained in a humidified atmosphere containing 5% CO_2_ at 37°C. For the incubation of cells under hypoxic states, cells were treated with serum-free media for 18 h and incubated in an airtight chamber (Thermo Forma Co., Marietta, OH, USA), which was flushed with a gas mixture containing 1% O_2_, 5% CO_2_ and 94% N_2_ for the indicated times at 37°C.

### Transfection experiments

For transfection, HCC (2 × 10^6^) were washed twice with PBS and then incubated in DMEM (10% FBS) with lentiviral vectors (5 μg) of pGFP-V-RS (shNC), shHIF1A, shp65, shc-Rel, mock, p65, cRel, miR-mock, miR-18a, miR-17, miR-199a-5p, or miR-93 (as listed in Supplementary Table 2) mixed with jetPRIME^®^ transfection reagent (10 μl) according to the manufacturer's instructions.

### Quantitative real-time PCR (qPCR)

Total RNA was isolated from cultured cells using TRIzol, and cDNA was synthesized with M-MLV reverse transcriptase according to the manufacturer's protocol. Expression was measured on an ABI Prism cycler (Applied Biosystems, Foster City, CA) and data were analyzed using the ΔΔCt method. The forward and reverse primers were listed in Supplementary Table 1. For mRNAs, *β*-actin served as internal control. Relative quantification of each miRNA was assessed using U6-2 as the housekeeping miRNA. After initial denaturation at 95°C for 1 min, PCR was performed for 30 cycles (30 s at 94°C, 1 min at 53°C and 2 min at 72°C) using Taq polymerase. Select reaction products (10 ml) were separated on 0.8% agarose gel, and stained with ethidium bromide. DNA band intensity was standardized and analyzed by densitometry using Phosphoimager and Quantity One software (Version 4.3.1) (Bio-Rad, Hercules, CA, USA).

### Western blot analysis

After various treatments, cells were washed once with ice-cold phosphate-buffered saline (PBS) and resuspended in 100–200 μl of lysis buffer (50 mM Tris, 150 mM NaCl, 5 mM ethylenediaminetetraacetic acid (EDTA), 5 mM EGTA, 1% sodium dodecyl sulfate (SDS), pH 7.5), then ultrasonicated on ice until the solution became clear. The total protein concentrations were measured *via* the Bradford method. The protein extracts (60 μg) were then subjected to SDS-PAGE electrophoresis and subsequent nitrocellulose membrane transfer. Western blots were probed overnight at 4°C, with specific primary antibodies in Tris-Buffered Saline Tween-20 (TBST) containing 5% skim milk. The primary antibodies used were HIF-1α (1:2000), HIF-1β (1:2000), p50 (1:1000), p65 (1:1000), c-Rel (1:1000), Dicer1 (1:1000), and *β*-actin (1:2000). After washing 3 times with TBST, the membranes were incubated for 1 h at room temperature with a respective conjugated IgG (1:20,000; Jackson ImmunoResearch Laboratories Inc., West Grove, PA, USA) in TBST containing 5% skim milk. Antigens were revealed using a chemiluminescence assay (Western Blotting Luminol Reagent, Pierce). Quantification of bands was achieved by densitometry using ImageJ software.

### miRNA sequences, targets, and gene nomenclature

The investigated miRNAs were reported in accordance with the miRBase registry gene annotation and nomenclature (http://www.mirbase.org/) or the National Center for Biotechnology Information (NCBI) database. Targets for select miRNAs were obtained with TargetScan (http://www.targetscan.org/) with the default parameters. The candidate miRNAs targeting the conserved 3′UTR of *HIF1A* were selected for c-Rel downstream miRNA prediction using miRGen (http://diana.cslab.ece.ntua.gr/mirgen/) with the default parameters.

### miRNA overexpression and 3′UTR experiment

miR-mock, miR-18a, miR-138, miR-199a-5p, or miR-93 was co-transfected with 3′UTR luciferase vectors of the vehicle control and *HIF1A* 3′UTR in HepG2 cells for 48 h. Luciferase activity was assayed using SuperLightTM Luciferase Reporter Gene Assay and measured using a Spectra Max M3 microplate reader (Molecular Devices, Inc., Sunnyvale, CA).

### ChIP assay

ChIP assay was performed using SABioscience kit following the manufacturer's instructions. Briefly, HepG2 and Huh7 cells treated with hypoxia for 4 h were washed with PBS and trypsinized. Chromatins in these cells (10^6^ cells per IP) were then cross-linked with 1% formaldehyde for 10 min at room temperature, sonicated to generate ~200- to 500-bp DNA fragments in lysis buffer, and immunoprecipitated with the indicated antibodies, or mouse IgG to capture the antibody-DNA complex. The enrichment of the eluted DNA was assessed by RT-PCR.

### Statistical analysis

Statistical analysis was performed using the statistical program SPSS 10.0 for Windows (SPSS Inc. Chicago, IL, USA). All data are presented as the mean ± S.E.M and were analyzed by one-way ANOVA. *P* values < 0.05 were considered to be statistically significant.

## SUPPLEMENTARY FIGURES AND TABLES


