# Supplementary material for: Temporal regulation of HIF-1 and NF-κB in hypoxic hepatocarcinoma cells
Source: Oncotarget. 2015 Mar 19;6(11):9409–19. doi: 10.18632/oncotarget.3352 (PMC4496226; doi:10.18632/oncotarget.3352)
Supplement: Supplementary file 1 [file oncotarget-06-9409-s001.pdf]

SUPPLEMENTARY FIGURES AND TABLES

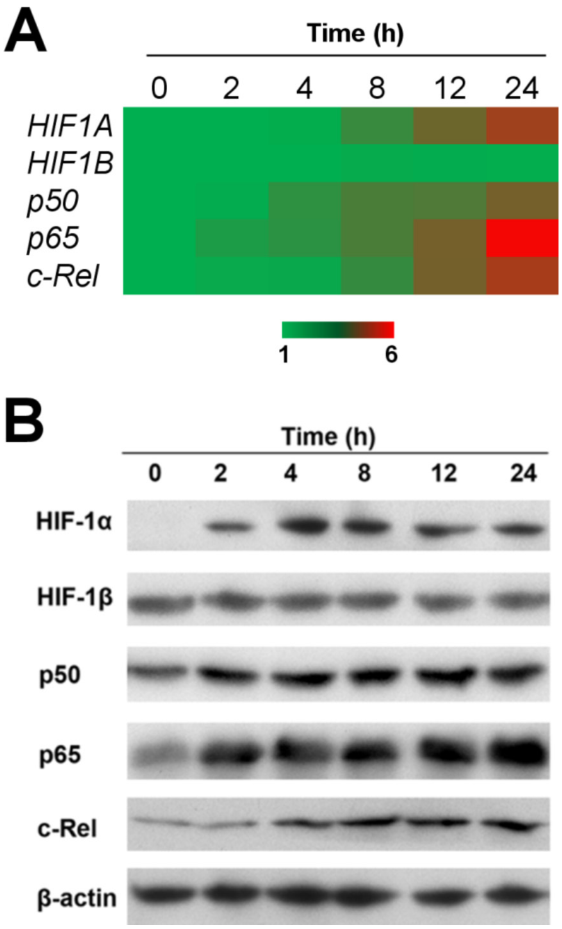

Supplementary Figure S1: Temporal expression of HIF-1α in Huh7 cells under short-term and prolonged hypoxia. Huh7 cells were exposed to 1% O_2 for 0–24 h and assessed the mRNA **(A)** and protein **(B)** expressions of HIF-1α, HIF-1β, NF-κB-p50, p65 and c-Rel. The results in (A) were expressed as the mean of n=4 independent experiments and expressed with heatmap.

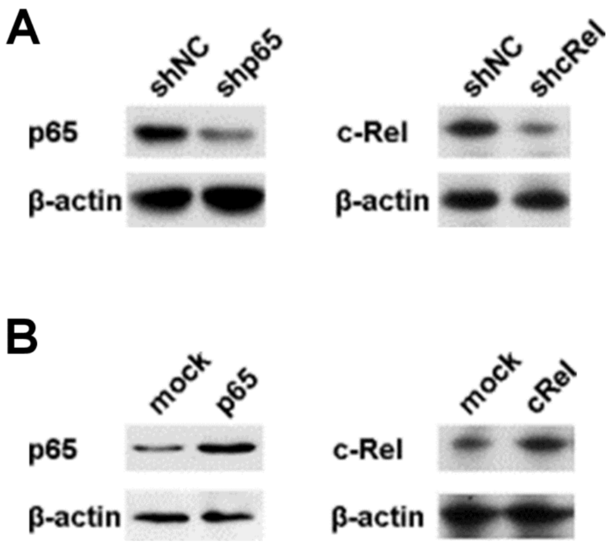

Supplementary Figure S2: Knockdown and overexpression of NF-κB in HepG2 cells. **(A)** HepG2 cells were transfected with shNC, shp65, or shcRel for 48 h. The protein expressions of p65, c-Rel and β-actin were determined with western-blot. **(B)** HepG2 cells were transfected with mock, p65, or cRel for 48 h. The protein expressions of p65, c-Rel and β-actin were determined with western-blot.

A

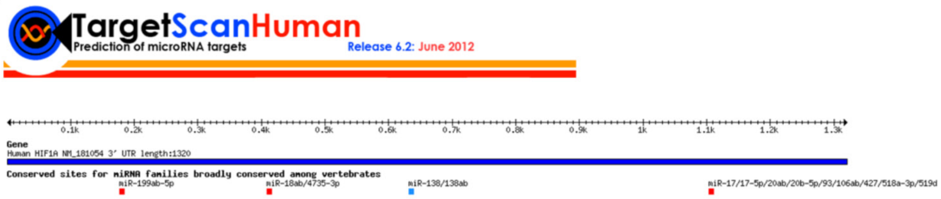

	predicted consequential pairing of target region (top) and miRNA (bottom)	seed match	site-type contri-bution	3' pairing contri-bution	local AU contri-bution	position contri-bution	TA contribution	SPS contribution	context+ score	context+ score percentile	conserved branch length	P_{CT}
Position 177-183 of HIF1A 3' UTR<br>hsa-miR-199a-5p	5' ...CAUUCCUUUUUUUGGACACUGGU...<br>3' CUUGUCCAUCAGACUUGUGACCC	7mer-m8	-0.120	0.021	0.007	-0.037	0.005	-0.032	-0.16	71	2.102	0.60
Position 177-183 of HIF1A 3' UTR<br>hsa-miR-199b-5p	5' ...CAUUCCUUUUUUUGGACACUGGU...<br>3' CUUGUCUAUCAGAUUUGUGACCC	7mer-m8	-0.120	0.030	0.007	-0.037	0.005	-0.032	-0.15	68	2.102	0.60
Position 409-415 of HIF1A 3' UTR<br>hsa-miR-4735-3p	5' ...AUCAUUUUAAAAAAUGCACCUUU...<br>3' UACAGAUUAAACUCGUGGAAA	7mer-m8	-0.120	-0.016	-0.144	-0.004	-0.016	-0.032	-0.33	94	2.012	0.58
Position 409-415 of HIF1A 3' UTR<br>hsa-miR-18a	5' ...AUCAUUUUAAAAAAUGCACCUUU...<br>3' GAUAGACGUGAUCUACGUGGAAU	7mer-m8	-0.120	0.012	-0.144	-0.004	-0.016	-0.032	-0.30	93	2.012	0.58
Position 409-415 of HIF1A 3' UTR<br>hsa-miR-18b	5' ...AUCAUUUUAAAAAAUGCACCUUU...<br>3' GAUUGACGUGAUCUACGUGGAAU	7mer-m8	-0.120	0.021	-0.144	-0.004	-0.016	-0.032	-0.30	92	2.012	0.58
Position 632-638 of HIF1A 3' UTR<br>hsa-miR-138	5' ...AUUUUCUUAAAAAAUACCAGCAG...<br>3' GCCGGACUAAGUGUUGUGGUCGA	7mer-1A	-0.074	0.001	-0.035	0.019	0.006	-0.066	-0.15	60	1.906	0.69
Position 1104-1110 of HIF1A 3' UTR<br>hsa-miR-20b	5' ...AAUGUUUGAUUUUAUGCACUUUG...<br>3' GAUGGACGUGAUACUCGUGAAAC	7mer-m8	-0.120	0.003	-0.075	-0.032	0.025	0.020	-0.18	90	1.735	0.62
Position 1104-1110 of HIF1A 3' UTR<br>hsa-miR-20a	5' ...AAUGUUUGAUUUUAUGCACUUUG...<br>3' GAUGGACGUGAUAUUCGUGAAAU	7mer-m8	-0.120	0.003	-0.075	-0.032	0.025	0.020	-0.18	90	1.735	0.62
Position 1104-1110 of HIF1A 3' UTR<br>hsa-miR-93	5' ...AAUGUUUGAUUUUAUGCACUUUG...<br>3' GAUGGACGUGCUUGUCGUGAAAC	7mer-m8	-0.120	0.021	-0.075	-0.032	0.025	0.020	-0.16	88	1.735	0.62
Position 1104-1110 of HIF1A 3' UTR<br>hsa-miR-17	5' ...AAUGUUUGAUUUUAUGCACUUUG...<br>3' GAUGGACGUGACAUUCGUGAAAC	7mer-m8	-0.120	0.021	-0.075	-0.032	0.025	0.020	-0.16	87	1.735	0.62
Position 1104-1110 of HIF1A 3' UTR<br>hsa-miR-106a	5' ...AAUGUUUGAUUUUAUGCACUUUG...<br>3' GAUGGACGUGACAUUCGUGAAAA	7mer-m8	-0.120	0.021	-0.075	-0.032	0.025	0.020	-0.16	87	1.735	0.62
Position 1104-1110 of HIF1A 3' UTR<br>hsa-miR-106b	5' ...AAUGUUUGAUUUUAUGCACUUUG...<br>3' UAGACGUGACAGUCGUGAAAU	7mer-m8	-0.120	0.021	-0.075	-0.032	0.025	0.020	-0.16	87	1.735	0.62
Position 1104-1110 of HIF1A 3' UTR<br>hsa-miR-519d	5' ...AAUGUUUGAUUUUAUGCACUUUG...<br>3' GUGAGAUUUCCCUCCGUGAAAC	7mer-m8	-0.120	0.040	-0.075	-0.032	0.025	0.020	-0.14	84	1.735	0.62

B

miRNA Symbol	Binding sequence	Distance from TSS
miR-199a-5p	tgGGGAActtcctc (NF-κB p50/p65)	-4785
	GGAAAtccca (c-Rel)	-3030
	gtggtCACGTgacaac (HIF-1)	-1549
miR-17	GGAAAtccca (c-Rel)	-2483
		-3098
miR-18a	GGAAAtccca (c-Rel)	-2483
		-3098
miR-20a	GGAAAtccca (c-Rel)	-2483
		-3098
miR-93	GGAAAtccca (c-Rel)	-36
		-2167
		-4313
miR-106b	GGAAAtccca (c-Rel)	-263
		-2394
		-4540

Supplementary Figure S3: HIF-1 upstream and c-Rel downstream miRNAs. **(A)** Targetscan predicted binding of miRNAs with conserved 3'UTR sites of *HIF1A*. **(B)** miRGen predicted c-Rel downstream and HIF-1 upstream miRNAs.

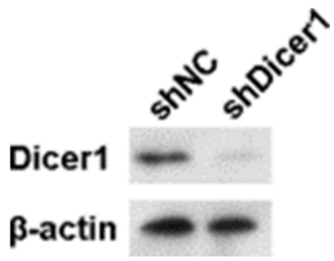

Supplementary Figure S4: Knockdown of Dicer1 in HepG2 cells. HepG2 cells were transfected with shNC or shDicer1 for 48 h. The protein expressions of Dicer1 and β-actin were determined with western-blot.

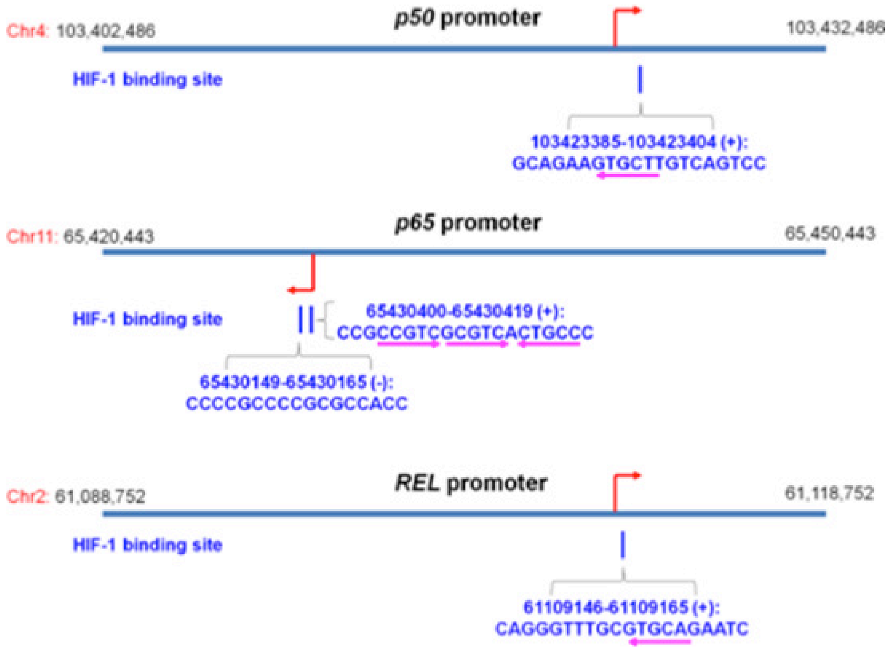

Supplementary Figure S5: HIF-1 binding site on NF-κB promoters. Vista predicted HIF-1 binding sites (blue) in the promoters of p50, p65, and c-Rel. Pink arrows show HRE sequences. Red arrow, transcription start site; +, positive strand.

Supplementary Table 1: Primers for target genes

Gene	Genebank ID	Forward	Reverse	Product Size
ACTB	NM_001101	catccgcaaagacctgtacg	cctgcttgctgatccacatc	218
HIF1A	NM_001243084	tccaagaagccctaacgtgt	tgatcgtctggctgctgtaa	180
ARNT/HIF1B	NM_001197325	aagccgtctttcctcactga	tgctgccaaaccattcagac	160
p50	NM_003998	aatggtggagtctgggaagg	tctgacgtttcctctgcact	226
p65	NM_001145138	acaagtggccattgtgttcc	acgtttctcctcaatccggt	169
cRel	NM_001291746	acctctgccttcttcaagct	gatgacgcttccattccgac	213
Dicer1	NM_001195573	tggtccacgagtcacaatca	cagccaatcgtacacagctc	209

	Qiagen Catlog No.
**U6-2**	MS00033740
**miR-199a-5p**	MS00006741
**miR-18a**	MS00031514
**miR-20a**	MS00003199
**miR-93**	MS00003346
**miR-17**	MS00029274
**miR-106b**	MS00003402

Supplementary Table 2: Plasmid vectors information

	Origene Catlog No.
**shNC**	TR30007
**shp65**	TL302038
**shcRel**	TL309876
**shHIF1A**	TG320380
**shDicer1**	TL308001
**mock**	PS100071
**p65**	RC220780L2
**cRel**	RC211878L2
	**ABM Catlog No.**
**mock-miR**	m003
**miR-93**	mh41079
**miR-199a-5p**	mh40255
**miR-17**	mh41055
**miR-18a**	mh41057
